# Dynamically Responsive Scaffolds from Microfluidic 3D Printing for Skin Flap Regeneration

**DOI:** 10.1002/advs.202201155

**Published:** 2022-06-02

**Authors:** Xiaocheng Wang, Yunru Yu, Chaoyu Yang, Luoran Shang, Yuanjin Zhao, Xian Shen

**Affiliations:** ^1^ Department of Gastrointestinal Surgery The First Affiliated Hospital Wenzhou Medical University Wenzhou 325035 China; ^2^ Department of Burns and Plastic Surgery Nanjing Drum Tower Hospital The Affiliated Hospital of Nanjing University Medical School Nanjing 210002 China; ^3^ Oujiang Laboratory (Zhejiang Lab for Regenerative Medicine, Vision and Brain Health) Wenzhou Institute University of Chinese Academy of Sciences Wenzhou Zhejiang 325001 China; ^4^ Shanghai Xuhui Central Hospital Zhongshan‐Xuhui Hospital and The Shanghai Key Laboratory of Medical Epigenetics The International Co‐laboratory of Medical Epigenetics and Metabolism (Ministry of Science and Technology) Institutes of Biomedical Sciences Fudan University Shanghai 200032 China

**Keywords:** 3D printing, microfluidics, photothermal, regeneration, scaffold, vascularization

## Abstract

Biological scaffolds hold promising perspectives for random skin flap regeneration, while the practical application is greatly limited by their insufficient vascularization ability and the lack of responsiveness during the dynamical healing process. Herein, a novel MXene‐incorporated hollow fibrous (MX‐HF) scaffold with dynamically responsive channels is presented for promoting vascularization and skin flap regeneration by using a microfluidic‐assisted 3D printing strategy. Benefiting from the photothermal conversion capacity of the MXene nanosheets and temperature‐responsive ability of poly(NIPAM) hydrogels in the MX‐HF scaffolds, they display a near‐infrared (NIR)‐responsive shrinkage/swelling behavior, which facilitates the cell penetration into the scaffold channels from the surrounding environment. Moreover, by incorporating vascular endothelial growth factor (VEGF) into the hydrogel matrix for controllable delivery, the MX‐HF scaffolds can achieve promoted proliferation, migration, and proangiogenic effects of endothelial cells under NIR irradiation. It is further demonstrated in vivo that the NIR‐responsive VEGF@MX‐HF scaffolds can effectively improve skin flap survival by promoting angiogenesis, decreasing inflammation, and attenuating apoptosis in skin flaps. Thus, it is believed that such responsive MX‐HF scaffolds are promising candidates for clinical random skin flap regeneration as well as other diverse tissue engineering applications.

## Graphical abstract

Microfluidic 3D printing of MXene‐incorporated hollow fibrous scaffolds with dynamically responsive channels is presented for promoting vascularization and skin flap regeneration. The MX‐HF scaffold displays a photothermal‐responsive shrinkage/swelling behavior under the control of NIR irradiation, which can facilitate the infiltration of cells or tissues into the scaffold channels and effectively improve skin flap regeneration.

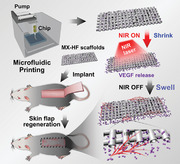

## 1 Introduction

Random skin flaps have been frequently used in orthopedic surgery for repairing or reconstructing tissue defects resulting from trauma, tumor resection, and congenital deformities.^[^ ^1^ ^]^ Unfortunately, the skin flap tissues commonly suffer from distal ischemic necrosis due to the insufficient blood supply and damaged homeostasis, particularly when the length‐to‐width ratio of the skin flaps exceeds 2:1.^[^ ^2^ ^]^ Therefore, skin flap survival is primarily restricted by adequate blood supply and good microcirculation. Specifically, the blood supply in random skin flaps even depends more on the subdermal vascularization than vascularization around the flaps.^[^ ^2^, ^3^ ^]^ Many approaches have been reported to increase blood supply and improve random skin flap survival, including the local administration of angiogenesis‐related growth factors such as the basic fibroblast growth factor and vascular endothelial growth factor (VEGF), or therapeutic drugs like vasodilators, antithrombotic agents and sympathetic blocking drugs.^[^ ^4^ ^]^ However, these treatments are limited by side effects of high‐dose usage, the short half‐life of the medicine, unsatisfactory efficacy, and tumorigenic risks. To address these issues, tissue engineering scaffolds have been previously utilized as delivery systems for various drugs and cytokines to improve vascularization and reduce necrosis in the skin flaps.^[^ ^3^, ^5^ ^]^ Moreover, these scaffolds with porous structures is conductive for cell ingrowth and substance exchange, which could improve skin flap survival by promoting vascularization and remodeling of local blood circulation systems. Despite some successes in enhanced vascularization and tissue regeneration, most of these scaffolds are with simple structures or morphologies, which cannot achieve the controllable and long‐term release of therapeutic agents. In addition, injured tissue regeneration is a dynamical and multifactorial process involving both intrinsic and environmental factors,^[^ ^6^ ^]^ while the dynamic interactions between the implanted scaffolds and surrounding tissues have been commonly ignored. Therefore, it is still highly desirable to develop an innovative scaffold with dynamically responsive structures and controllable release capability to improve skin flap survival.

**Figure 1:**
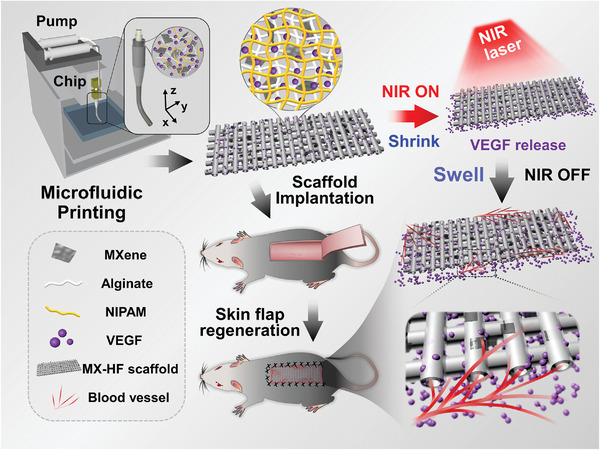
Schematic illustration of the dynamically responsive scaffolds from microfluidic 3D printing for skin flap regeneration. The MX‐HF scaffolds can be fabricated via a coaxial capillary microfluidic strategy. The MX‐HF scaffolds displays a photothermal‐responsive shrinkage/swelling behavior under the control of NIR irradiation, which can facilitate the infiltration of cells or tissues into the scaffold channels. In addition, the controllable VEGF release from the dynamic channeled scaffolds can exert promotive effects on the formation of new blood vessels and large‐scaled skin flap regeneration.

Herein, we propose a novel MXene‐incorporated hydrogel scaffold with the desirable features for skin flap regeneration by using a microfluidic‐assisted 3D printing strategy, as schemed in **Figure** **1**. Microfluidic technology can manipulate fluids in single or multiple channels with dimensions from tens to hundreds of microns.^[^ ^7^ ^]^ Thus, the integration of microfluidic systems with conventional extrusion‐based 3D printing platforms enables the precise control of the compositional and structural properties of tissue engineering scaffolds such as biomimetic vascular‐like channeled scaffolds.^[^ ^8^ ^]^ These microfluidic 3D printing scaffolds have been employed in tissue engineering,^[^ ^9^ ^]^ and their vascular‐like biomimetic channel structures have been demonstrated specifically effective for promoting the formation of 3D vascular networks and regeneration of new tissues.^[^ ^8c^ ^]^ However, their values in skin flap regeneration are still challenging due to its rigorous regeneration demand. In contrast, MXene is known as a new class of 2D transition metal materials that have found broad applications in many fields, including wearable electronics, energy storage, catalysis, biomedical applications, etc.^[^ ^10^ ^]^ Notably, MXene nanosheets have attracted wide interest in constructing intelligent drug delivery systems regarding their unique photothermal properties and biodegradable performance.^[^ ^11^ ^]^ Therefore, we envision that the MXene‐incorporated channeled scaffolds with photothermal responsive features could be easily fabricated via the microfluidic 3D printing strategy, and their channel structures and controllable release capability is expected to greatly facilitate the vascularization and improve skin flap survival.

In this manuscript, we present a capillary‐assembled microfluidic printing strategy to prepare the desired MX‐HF scaffolds for promoting vascularization and skin flap regeneration (Figure 1). The MX‐HF scaffolds were generated by polymerizing the mixture fluid of MXene (Ti_3_C_2_) nanosheets, *N*‐isopropylacrylamide (NIPAM), and alginate in the microfluidic coaxial channels, which was then acted as the printable bio‐ink for the 3D scaffold creation. Owing to the photothermal conversion capacity of the MXene nanosheets and temperature‐responsive ability of NIPAM polymers, the as‐designed MX‐HF scaffold exhibited a near‐infrared (NIR)‐responsive shrinkage/swelling behavior, which could facilitate cell enrichment within scaffold channels. Additionally, the controllable delivery capacity of MX‐HF scaffolds could be realized by incorporating VEGF into the scaffold matrix, contributing to the promoted migration, proliferation, and proangiogenic effects of human umbilical vein endothelial cells (HUVECs) under NIR irradiation. Thus, it was demonstrated through an in vivo study that the NIR‐responsive VEGF@MX‐HF scaffolds could effectively improve skin flap survival by promoting angiogenesis, decreasing inflammation, and attenuating apoptosis in the skin flaps. These results indicate that such MX‐HF scaffolds are ideal candidates for clinical random skin flap regeneration.

## 2 Results and Discussion

### 2.1 Characterization of MX‐HF Scaffolds

In the present study, the MX‐HF scaffolds were fabricated using a microfluidic‐assisted bioprinting method (Figure 1). First, a coaxial capillary microfluidic chip was custom‐made by coaxially inserting a spindle capillary (orifice diameter: 100 µm) into a tapered capillary (orifice diameter: 450 µm; Figure S1, Supporting Information). During microfluidic spinning, the MX‐HF microfibers with straight hollow channels could be generated via ionic crosslinking between the Ca ions in the poly(vinyl alcohol) (PVA) inner fluid and the alginate in the MXene‐containing outer fluid. As shown in Figure S2 (Supporting Information), the outer diameter of the microfibers increased from 450 to 550 µm by increasing the inner flow rate ranging from 0.02 to 0.36 mL h^−1^ or the outer flow rate ranging from 1 to 6 mL h^−1^. Comparatively, the increased inner flow rate brought about the increased channel diameter from 90 to 270 µm, while the increased outer flow rate led to the decreased inner channel diameter from 250 to 100 µm. When the inner and outer flow rates were set to 0.2 and 3 mL h^−1^, a typical hollow microfiber with a straight channel was obtained, whose outer and inner diameters were approximately 500 and 200 µm, respectively (Figure S3a–c, Supporting Information). By introducing the MXene nanosheets (Figure S4, Supporting Information) into the NIPAM‐based biopolymers, MXene‐containing microfibers could be generated with nanosheets randomly distributed in the biopolymer struts, distinct from the pure microfibers without MXene nanosheets (Figure S3d–i, Supporting Information).

**Figure 2:**
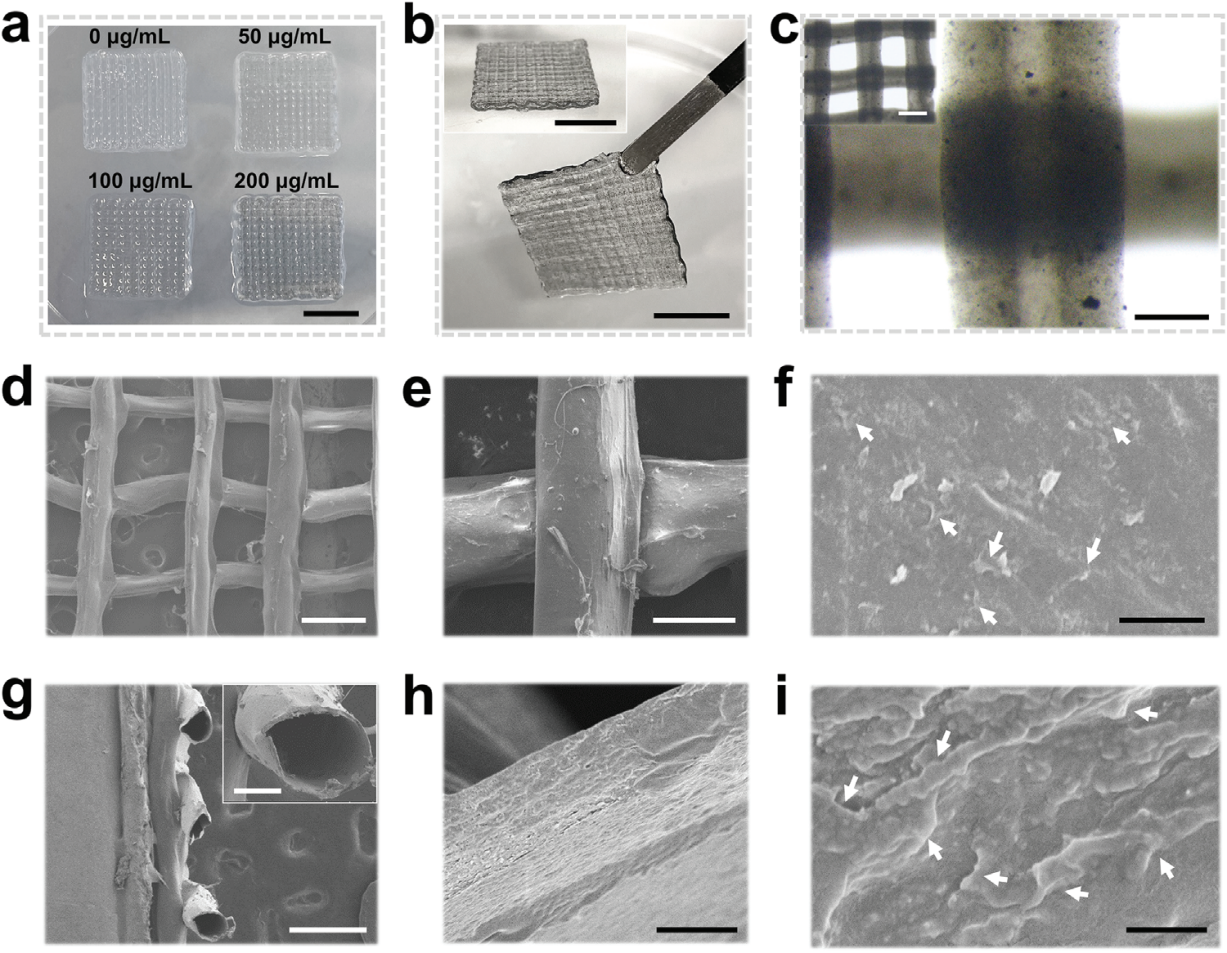
Characterization of MX‐HF scaffolds. Photographs of a) MX‐HF scaffolds with different MXene contents (MXene concentration in the hydrogel precursor: 0, 50, 100, and 200 µg mL^−1^). Scale bar indicates 1 cm. b) Photographs of the different views of the hollow fibrous scaffolds (MXene content: 200 µg mL^−1^). Scale bars indicate 1 cm. c) Optical micrographs of the hollow scaffolds with straight channels. Scale bars indicate 200 and 500 µm (inset). d–f) Top and g) section views of the scanning electron microscope (SEM) images of the freeze‐dried MX‐HF scaffolds at different magnifications. h,i) The high‐resolution SEM images of the scaffold channels indicated the incorporated MXene nanosheets within the MX‐HF scaffolds. The white arrows in (f) and (i) indicate the nanosheets incorporated in the scaffold matrix. Scale bars indicate 500 µm in (d) and (g), 150 µm in (e), 100 µm in (g) (inset), and 5 µm in (h), 2 µm in (f), and 500 nm in (i).

Subsequently, the coaxial microfluidic chip was used to replace the original printing nozzle in a programmable 3D printer. When the flow rate of the fluids well matched the moving speed of the printing head, the MXene‐containing microfibers could be layer‐stacked into a 3D scaffold in a Petri dish containing 0.08% CaCl_2_ and 30% ethanol. The appearance of MX‐HF scaffolds gradually changed from light white to dark gray as the MXene contents increased (**Figure** **2**a). The printed MX‐HF scaffolds could be easily picked up with tweezers and maintained their structural stability after further ionic crosslinking in 2% CaCl_2_ and photopolymerization by UV irradiation (Figure 2b). As the diffusion rate of Ca ions in biopolymer fluids was slower than the printing speed during printing, the solidification of the MXene‐containing microfibers was incomplete when layer‐stacked into 3D constructs in the low‐concentration CaCl_2_ bath, which contributed to the good connections of the fibrous struts with straight channels (Figure 2c; Figure S5a–f, Supporting Information).

The microstructure of the obtained MX‐HF scaffolds was visualized under a scanning electron microscope (SEM; Figure 2d–i). As compared to the hollow fibrous (HF) scaffolds without MXene nanosheets (Figure S5g–i, Supporting Information), there are many nanosheets randomly distributed in the hollow channels of MX‐HF scaffolds, confirming the successful incorporation of MXene nanosheets into the scaffold matrix. Both the 3D‐printed HF and MX‐HF scaffolds displayed interconnected macropore structures (≈500 µm; as shown in Figure 2c and Figure S5 in the Supporting Information), which is beneficial for cell infiltration and efficient exchange of nutrients. Additionally, the programmable printing platform enabled the fabrication of MX‐HF scaffolds with larger sizes (Figure S6, Supporting Information), which are highly desirable to match the large‐scaled tissue defects in skin flap regeneration applications. Taking together, the above results demonstrated the feasibility of the microfluidic‐assisted bioprinting strategy for fabricating MX‐HF scaffolds with controllable MXene contents and hollow channels.

### 2.2 Photothermal Responsiveness of MX‐HF Scaffolds

**Figure 3:**
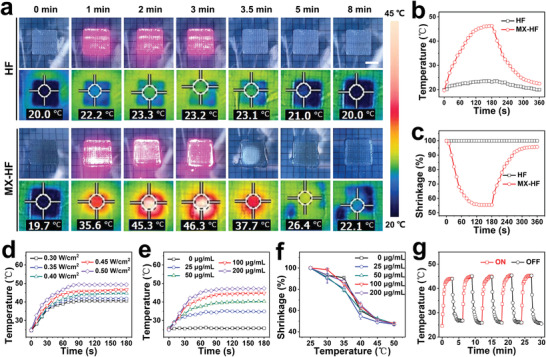
Photothermal responsive performance of the MX‐HF scaffolds. a) Real‐time photographs and thermal images, corresponding b) photothermal heating curves and c) volume shrinkage ratio of HF and MX‐HF scaffolds when exposed to NIR laser irradiation at 0.40 W cm^−2^ for 3 min (ON), and followed by naturally cooling to room temperature without irradiation for 3 min (OFF). Scale bar indicates 1 cm. Photothermal heating curves of d) MX‐HF scaffolds with MXene contents of 100 µg mL^−1^ at different laser power densities of 0.30, 0.35, 0.40, 0.45, and 0.50 W cm^−2^ for 3 min and e) MX‐HF scaffolds with different MXene contents (MXene concentration: 0, 25, 50, 100, and 200 µg mL^−1^) under continuous 808 nm irradiation at a power intensity of 0.40 W cm^−2^ for 3 min. f) Volume shrinkage ratio of MX‐HF scaffolds with different MXene contents at different temperatures (*n* = 4 per group). g) Temperature variation over five ON (red line)/OFF (black line) cycles of NIR irradiation (0.40 W cm^−2^, 2 min).

Due to the high NIR photothermal conversion property of MXene nanosheets, the obtained MX‐HF scaffolds are expected to be responsive to NIR irradiation. The real‐time volume changes and temperature variation of HF and MX‐HF scaffolds were recorded under the irradiation of a NIR laser (808 nm, 0.45 W cm^−2^, 3 min, laser ON), which was then turned off for natural cooling (laser OFF, **Figure** **3**a–c). The MX‐HF scaffolds showed a sharp temperature increase from 19.7 to 46.3 °C, while the HF scaffolds without MXene nanosheets did not show obvious photothermal effect. More importantly, the MX‐HF scaffolds shrank dramatically when the temperature exceeds 35 °C after 1 min NIR treatment, which could be attributed to the temperature‐responsive volume phase change of poly(NIPAM) hydrogels. It is well known that poly(NIPAM) is a thermosensitive hydrogel with reversible volume phase transition ability in response to the surrounding temperature. As demonstrated in our previous works, the poly(NIPAM)‐based hydrogels could shrink slightly above the physiological temperature, and swell after cooling down when copolymerizing the NIPAM with *N*‐methylol acrylamide.^[^ ^8^, ^12^ ^]^ Our present results further verified the reversible shrinkage and swelling behavior of MX‐HF scaffolds under the control of NIR irradiation. In detail, a 55% volume shrinkage of the MX‐HF scaffold was found when the temperature exceeded 40 °C, and the volume recovered to 95% when cooling down to around 20 °C within 3 min. In addition, the NIR‐induced temperature increase of MX‐HF scaffolds could be effectively controlled from 20 to 50 °C by changing the laser power densities from 0.30 to 0.50 W cm^−2^ and MXene contents from 0 to 200 µg mL^−1^ (Figure 3d,e). More importantly, the MX‐HF scaffolds maintained their photothermal sensitivity regardless of the variation of MXene concentrations, and all the MX‐HF scaffolds shrank dramatically when the temperature exceeds 35 °C (Figure 3f). Furthermore, the photothermal stability of MX‐HF scaffolds was confirmed by the negligible changes of temperature profiles upon NIR irradiation for five ON/OFF cycles (Figure 3g). Collectively, these data indicated the MX‐HF scaffolds with controllable and repeatable NIR responsive capacity, allowing for the dynamic interactions with surroundings and regulation of microcirculation in practical tissue engineering applications.

### 2.3 In Vitro Regenerative Activity of the MX‐HF Scaffolds

**Figure 4:**
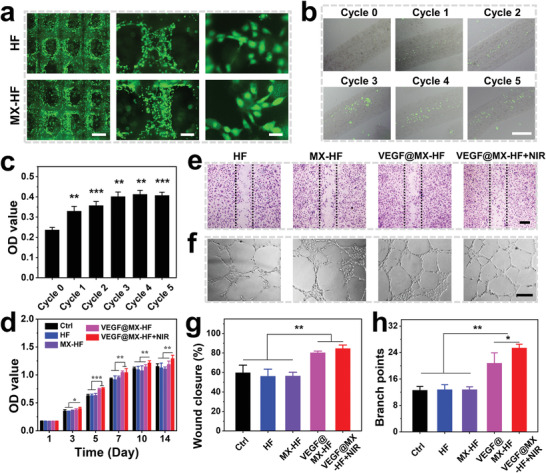
In vitro regenerative properties of the NIR‐responsive dynamic scaffolds. a) Live/dead staining of human umbilical vein endothelial cells (HUVECs) adhered to the surface of HF and MX‐HF scaffolds for 24 h. Alive or dead cells were in green or red, respectively. Scale bars indicate 1 mm, 300 µm, and 50 µm from left to right. b) Fluorescent images and c) CCK8 assay of HUVECs enriched in the MX‐HF channels after NIR irradiation (0.40 W cm^−2^, 2 min for each cycle). Scale bar indicates 200 µm. d) Cell proliferation of HUVECs cultured with different scaffolds for 14 days. e,f) Representative images of e) in vitro scratch assay and f) Matrigel tube formation of HUVECs cultured with different scaffolds. Dotted lines in (e) indicate initial scratch edges. Scale bars indicate 300 µm in (e), and 200 µm in (f). g,h) Quantification of g) closure rates for scratch assay after 15 h and h) Matrigel tube formation after 6 h of HUVECs. *n* = 6 per group, two‐tailed unpaired Student's *t*‐tests were performed to calculate the statistical significance between two groups, and *p < 0.05, **p < 0.01, and ***p < 0.001.

The in vitro regenerative potential of the MX‐HF scaffolds was investigated by culturing HUVECs with different scaffolds. HUVECs were seeded on the HF and MX‐HF (MXene content: 100 µg mL^−1^) scaffolds. The morphology of cells adhered to the scaffold surface was visualized using live/dead staining after the incubation of 24 h. All cells were alive as indicated by the strong green fluorescence and well spread on the scaffold struts (**Figure** **4**a). Benefiting from the NIR‐responsive swelling/shrinkage features, the surrounding cells could be indrawn to the scaffold channels by controlling the “ON/OFF” cycles of an 808 nm laser. The MX‐HF channels would shrink when the NIR laser was “ON,” and expand when the laser was “OFF.” Such reversible swelling/shrinkage behaviors allowed the infiltration of more cells into the scaffold channels via increasing “ON/OFF” cycles, even in the deep regions (Figure 4b,c). After the repeated treatments of NIR‐induced mild hyperthermia (≈40 °C), the cells inside the MX‐HF channels could maintain their proliferation activity when continuously cultured for 3 days (Figure S7, Supporting Information).

Furthermore, the NIR‐responsive swelling/shrinkage features endowed the MX‐HF scaffolds with controllable drug‐releasing ability, which was subsequently tested by incorporating VEGF in MX‐HF scaffolds for the enhanced in vitro regenerative capacity and controllable VEGF release (Figure S8, Supporting Information). Owing to the released VEGF from the VEGF@MX‐HF scaffolds, the cell proliferation of HUVECs was significantly promoted in the VEGF@MX‐HF and VEGF@MX‐HF+NIR groups as compared to the MX‐HF and HF groups (Figure 4d; Figure S9, Supporting Information). On days 10 and 14, the OD value for HUVECs in the VEGF@MX‐HF+NIR group was significantly higher than the VEGF@MX‐HF group, indicating the enhanced cellular metabolism by NIR‐responsive VEGF@MX‐HF scaffolds. To explore whether the VEGF@MX‐HF scaffolds could promote cell migration under NIR irradiation, a typical scratch assay was conducted. The results revealed that the wound closure of HUVECs after 15 h was significantly accelerated in the VEGF@MX‐HF groups in comparison to the other groups (Figure 4e,g). To evaluate the angiogenic capability of NIR‐responsive dynamic scaffolds, a Matrigel tube formation assay was performed and the results showed that more vessel‐like tubes were observed after 6 h in the VEGF@MX‐HF+NIR groups than those in other groups (Figure 4f,h). Altogether, these results demonstrated the NIR‐enhanced cell penetration and controllable drug delivery capacity of the dynamic MX‐HF scaffolds, which were expected to facilitate the formation of blood vessels and ingrowth of new tissues after scaffold implantation in vivo.

### 2.4 In Vivo Regenerative Activity of the Dynamical Scaffolds

**Figure 5:**
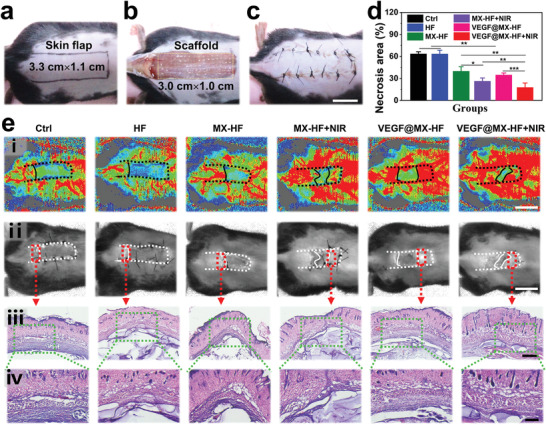
The skin flap survival rates after treatment. a) A random skin flap animal model, in which the flap was 1.1 cm (width) × 3.3 cm (length) on the mouse dorsal side with the pedicle at the tail end. b) After flap elevation, an MX‐HF scaffold with 1.0 cm (width) × 3.0 cm (length) × 0.5 mm (height) was implanted into the flap skin. c) The skin flaps were then sutured back in situ using silk sutures. Scale bar indicates 1 cm. d) Flap necrosis area percentages of different groups on day 9. *n* = 6 per group, two‐tailed unpaired Student's *t*‐tests were performed to calculate the statistical significance between two groups, and *p < 0.05, **p < 0.01, and ***p < 0.001. e) Histological analysis of the necrotic junction of the skin flaps in different groups: i) The real‐time blood flow images captured with laser speckle contrast imaging; ii) corresponding photos of skin flaps; iii,iv) H&E staining of the necrosis and survival junction area of the skin flaps in different groups. Scale bars indicate 1 cm in (i) and (ii), 500 µm in (iii), and 200 µm in (iv).

The ability of the dynamical MX‐HF scaffolds to promote vascularization and tissue regeneration was further evaluated using an autologous skin transplantation model in mice. As shown in **Figure** **5**a–c, a random skin flap of 1.1 cm × 3.3 cm was elevated and an HF, MX‐HF or VEGF@MX‐HF scaffold of 1.0 cm × 3.0 cm was subcutaneously implanted before the reposition of the skin flap. To trigger the reversible swelling/shrinkage behavior of MX‐HF and VEGF@MX‐HF scaffolds, NIR irradiation (0.50 W cm^−2^, 3 min ON/OFF cycles for five times) was performed every three days after the scaffold implantation. The temperature in the skin flaps with MX‐HF and VEGF@MX‐HF scaffolds implanted rapidly reached approximately 45 °C within 3 min (Figure S10, Supporting Information). Macroscopically, the survival skin flaps were obviously different from the necrotic ones, which gradually turned into stiff and dark. As shown in Figure S11a (Supporting Information), the skin flap necrosis without scaffold implantation (i.e., control group) was remarkably clearer as compared to all scaffold‐treated mice. The necrosis of skin flaps in groups was quantified on day 9. The necrosis rates were 63.7 ± 2.7% and 63.5 ± 5.1% in the control and HF groups, respectively, while the average necrosis rates were 40.0 ± 5.9% and 34.9 ± 2.6% in the MX‐HF and VEGF@MX‐HF groups, respectively (Figure 5d). By comparison, the necrosis rates were significantly lower in the skin flaps treated with NIR‐irradiated MX‐HF and VEGF@MX‐HF scaffolds, whose average necrosis rates were 26.3 ± 4.3% and 17.9 ± 5.9%, respectively. These results indicated that the mild NIR treatment showed no obvious harm to the skin flaps, but instead promoted the skin flap survival, probably because of the NIR‐induced dynamic behaviors of the MX‐HF scaffolds.

To investigate the effect of different scaffolds on the blood supply of the skin flaps, the real‐time blood flow of the skin flaps was measured using a laser Doppler perfusion imager on day 9. As displayed in Figure 5ei,ii, the NIR‐irradiated MX‐HF or VEGF@MX‐HF scaffolds could remarkably increase the vascularization and survival rates of the skin flaps, in contrast to the less blood flow in the MX‐HF or VEGF@MX‐HF groups without irradiation, indicating that NIR‐induced dynamic scaffolds were superior to static scaffolds in promoting blood perfusion. The angiogenesis states of detached skin flaps were examined under optical microscopy (Figure S11b, Supporting Information). It was found that much more 3D vascular networks and new tissues could be clearly observed under the skin flaps from the VEGF@MX‐HF+NIR group than other groups, which was potentially beneficial for the skin flap survival. Furthermore, H&E and Masson staining were performed on the necrotic junction of the skin flaps. As shown in Figure 5eiii,iv and Figure S12 (Supporting Information), intact epithelial structures, abundant granulation tissues, and neatly arranged collagen fibers around the VEGF@MX‐HF scaffolds were observed in the skin flaps, verifying the improved survival rates of random skin flaps by such dynamic NIR‐responsive scaffolds.

**Figure 6:**
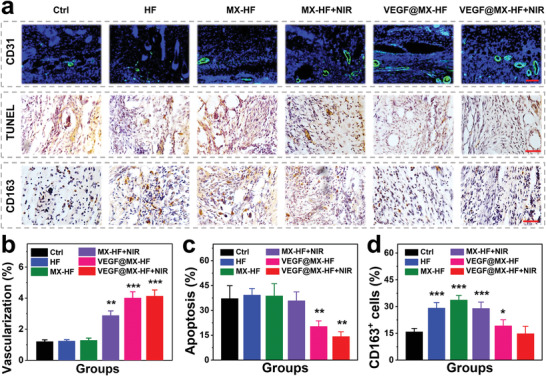
Angiogenesis, apoptosis and inflammation in skin flaps. a) Immunohistochemical staining images of skin flaps with anti‐CD31 antibodies (green) and DAPI (blue), TUNEL staining and CD163 staining in different groups. Scale bars indicate 100 µm (top) and 50 µm (middle, bottom). Quantification of the b) CD31 positive vessels, c) cell apoptosis assessed by TUNEL staining and d) CD163 positive macrophages. *n* = 6 per group, two‐tailed unpaired Student's *t*‐tests were performed to calculate the statistical significance between two groups, and *p < 0.05, **p < 0.01, and ***p < 0.001 compared with the control group.

Immunohistochemical analysis was conducted to further investigate the angiogenesis, apoptosis and inflammation response of the scaffolds in the skin flaps (**Figure** **6**). The skin flap neovascularization was assessed using CD31 immunofluorescence staining. More microvessels were observed in the VEGF@MX‐HF treated groups, while there is no significant difference among the control, HF and MX‐HF groups (Figure 6a), which was mainly attributed to the vascularization ability of VEGF incorporated in the scaffolds. Quantitatively, the average microvessel densities were 1.2 ± 0.1%, 1.2 ± 0.1%, 1.3 ± 0.1%, 2.9 ±0.3%, 4.0 ± 0.4%, and 4.1 ± 0.4% in the control, HF, MX‐HF, MX‐HF+NIR, VEGF@MX‐HF, and VEGF@MX‐HF+NIR groups, respectively (Figure 6b). Insufficient blood supply may contribute to cell apoptosis in the distal part of the skin flaps. Thus, TUNEL staining was employed to explore the protective effect of the VEGF@MX‐HF scaffolds against cell apoptosis. Fewer TUNEL‐positive cells were found in the VEGF@MX‐HF (20.4 ± 3.3%) and VEGF@MX‐HF+NIR (14.2 ± 2.8%) groups than those in the control (37.2 ± 7.6%), HF (39.2 ± 3.8%), MX‐HF (38.8 ± 7.3%), and MX‐HF+NIR (35.9 ± 5.2%) groups (Figure 6a,c). Apart from angiogenesis and apoptosis, local inflammation also plays a crucial role in skin flap regeneration as the blood supply is restricted. Excess inflammation could cause damage and even necrosis to flap tissues in continuous inflamed state because of the ischemia/reperfusion injury. Therefore, macrophage infiltration was detected by CD163 immunohistochemical staining to assess the inflammation response of the implanted scaffolds in the skin flaps. Figure 6a,d shows lower densities of CD163 positive cells in the VEGF@MX‐HF (19.3 ± 3.3%) and VEGF@MX‐HF+NIR (14.9 ± 4.0%) groups than those in the HF (29.2 ± 3.0%), MX‐HF (33.8 ± 2.5%), and MX‐HF+NIR (29.0 ± 3.5%) groups, probably because the VEGF release from the MX‐HF scaffolds could promote vascularization and thereby alleviate the local inflammatory reaction in skin flaps. Besides, the less CD163 positive cells appeared around the implanted VEGF@MX‐HF scaffolds also implied that the good histocompatibility of VEGF@MX‐HF scaffolds would not induce extensive inflammation.

## 3 Conclusion

In summary, a microfluidic‐assisted printing strategy has been proposed to prepare the dynamically responsive channeled scaffolds for improving vascularization and promoting random skin flap survival. Unlike the bulk hydrogels or electrospun membranes previously reported in skin flap regeneration,^[^ ^2^, ^3^, ^4^ ^]^ the present work developed a dynamic hydrogel scaffold with highly ordered 3D macropores to promote vascularization for skin flap regeneration. More importantly, the scaffolds were featured with NIR‐responsive reversible shrinkage/swelling performance, which greatly facilitated the cellular infiltration into the scaffold channels. Moreover, the in vitro regenerative capacity of MX‐HF scaffolds could be enhanced by incorporating VEGF into the scaffold matrix, as demonstrated by the promoted cell proliferation, migration, and proangiogenic effects in vitro. Finally, the in vivo tissue regenerative efficacy of the dynamic scaffolds was confirmed to enhance skin flap survival rates by promoting angiogenesis, reducing apoptosis, and decreasing inflammation in skin flaps. Therefore, such dynamically responsive scaffolds could be effective, safe and easily applicable candidates for the promotion of rapid vascularization and skin flap survival, representing bright prospects in regenerative medicine, and other tissue engineering fields.

## 4 Experimental Section

### Materials

Isopropylacrylamide (NIPAM) was purchased from Shanghai Macklin. MXene (Titanium Carbide, Ti_3_C_2_) was purchased from 11 Technology. Calcium chloride (CaCl_2_) and sodium alginate were bought from Alfa Aesar. Poly(vinyl alcohol) (PVA, low viscosity), *N*‐*N*‐methylol acrylamide (NMAM), lithium phenyl‐2,4,6‐trimethylbenzoylphosphinate (LAP), *N*,*N*′‐methylenebis (acrylamide) (BIS) were obtained from Shanghai Aladdin. Growth Factor Reduced Matrigel was obtained from Corning Life Sciences. Recombinant human vascular endothelial growth factor 165 (VEGF‐165), human VEGF enzyme‐linked immunosorbent assay (ELISA) kit, and Cell Counting Kit‐8 (CCK8) were bought from Beyotime Biotechnology. Human umbilical vein endothelial cells (HUVECs) and specified endothelial cell medium (ECM) were purchased from ScienCell. The live/dead, hematoxylin–eosin (H&E) and Masson's trichrome staining kits were purchased from Beijing Solarbio. Deionized water (18.2 MΩ cm, 25 °C) was produced using a Millipore purification system.

### Microfluidic Spinning of MXene‐Containing Hollow Microfibers

A coaxial capillary microfluidic chip was custom‐made to generate the MXene‐containing hollow microfibers. Typically, a glass capillary with a spindle tip (orifice diameter: 100 µm) was inserted into a capillary with a tapered tip (orifice diameter: 450 µm). The two coaxial glass capillaries were stuck to a piece of glass slide with transparent epoxy resin (Figure S1, Supporting Information). The inner fluid was 10% PVA solution containing 0.8% CaCl_2_, and the outer fluid was the mixture fluid containing NIPAM (15% w/v), NMAM (1.5% w/v), sodium alginate (2.5% w/v), Matrigel (20% v/v), BIS (0.5% w/v), LAP (0.1% v/v), and a certain amount of MXene nanosheets (0–200 µg mL^−1^). Both the inner and outer liquids were injected into the microfluidic chip using programmed syringe pumps with defined flow rates. The hollow channels were formed by primarily ionic crosslinking between the inner Ca ions and outer alginate biopolymers. The stable gelation of the MXene‐containing microfibers was obtained from the subsequent photopolymerization of NIPAM components via UV irradiation and ionic crosslinking of alginate components in 2% CaCl_2_. The microfibers were collected in a glass vessel containing 2% CaCl_2_ and further photo‐crosslinked under UV irradiation. Different MXene‐containing microfibers could be obtained by varying MXene concentrations in the outer pre‐gel solutions with 0, 25, 50, 100, and 200 µg mL^−1^.

### Microfluidic Printing of MXene‐Containing Hollow Fibrous Scaffolds

A microfluidic‐assisted 3D printing method was employed to fabricate the MX‐HF scaffolds. The custom‐made coaxial microfluidic chip was used to replace the original nozzle in a programmable 3D printer. The scaffold with the designed size and shape was transferred as the 3D model for printing. During microfluidic printing, the nozzle moving speed was adjusted to match the extrusion of the hollow microfibers, which were stacked to a 3D constructs layer by layer in a Petri dish containing 0.08% CaCl_2_ and 30% ethanol. Typically, the moving speed of the printer nozzle was set to 6 mm s^−1^, while the outer and inner flow rates of phases were set to 3 and 0.2 mL h^−1^, respectively. The scaffold channels formed because of the ionic crosslinking between the Ca ions diffused from the inner flow phase and the alginate component in the outer flow phase. Stable gelation of the 3D scaffolds was achieved after printing by further photopolymerization of the NIPAM component by UV irradiation and ionic crosslinking of the alginate component in 2% CaCl_2_. MX‐HF scaffolds with different MXene contents could be obtained by varying MXene concentrations in the outer pre‐gel solutions with 0, 25, 50, 100, and 200 µg mL^−1^. VEGF incorporated MX‐HF (VEGF@MX‐HF) scaffolds were obtained by adding the VEGF‐165 (concentration: 20 ng mL^−1^) in the outer pre‐gel solutions. All reagents and liquids were sterilized by UV irradiation overnight or filtration through a sterile 0.22 mm filter.

### Characterizations

Optical bright‐field and fluorescent images were obtained via an inverted fluorescence microscope (ZEISS Axio Vert. A1, Germany) or a stereomicroscope (Olympus BX51, Tokyo, Japan). The microstructure of the freeze‐dried MX‐HF scaffolds was visualized using a scanning electron microscope (SEM, SU8010, Hitachi, Japan).

### Photothermal Performance of MXene‐Containing Fibrous Scaffolds

HF and MX‐HF (MXene concentration: 200 µg mL^−1^, size: 20 mm × 20 mm × 2 mm) scaffolds were exposed to the NIR irradiation (808 nm, size: 20 mm × 20 mm, 3 min; Figure 3a). The temperature and volume changes of the scaffolds were monitored in real‐time. The corresponding thermal images were recorded using an infrared thermal camera (FLIR E5‐XT, Germany). The photothermal responsiveness of MX‐HF scaffolds were tested with different MXene contents in the range of 0–200 µg mL^−1^ under the 808 nm irradiation with varied power densities in the range of 0.30–0.50 W cm^−2^. The volume changes of MX‐HF scaffolds with different MXene contents were tested at different temperature. The scaffold shrinkage rate was calculated using the following formula: Scaffold shrinkage (%) = *V*/*V*_0_ × 100%. *V* represents the volume of MX‐HF scaffolds after NIR irradiation and *V*_0_ represents the volume of MX‐HF scaffolds before NIR irradiation. To explore the photothermal stability of the MX‐HF scaffolds, they were treated with 808 nm laser irradiation at 0.40 W cm^−2^ for 2 min and subsequently underwent natural cooling for 2 min. The procedure was repeated 5 times (i.e., five 2 min ON/OFF cycles).

### Cell Adhesion and Proliferation Assay

2 × 10^5^ of HUVECs were seeded on the HF or MX‐HF scaffold (MXene concentration: 100 µg mL^−1^; size: 20 mm × 20 mm × 2 mm) and cultured at 37 °C, 5% CO_2_. For visualizing the cells adhered to the scaffold surface, the cells were stained using a live/dead kit after incubation for 24 h. For cell proliferation assay, 5 × 10^3^ of HUVECs were cultured in 24‐well plates with the HF, MX‐HF, or VEGF@MX‐HF scaffold for 14 days. The release amounts of VEGF from the VEGF@MX‐HF scaffolds were detected using VEGF ELISA kits every day after NIR irradiation (808 nm, 0.40 W cm^−2^, 3 min). The NIR‐induced temperature was kept around 45 ℃. CCK8 assay was performed on days 1, 3, and 5.

### NIR‐Enhanced Cell Infiltration into the Scaffold Channels

The channeled microfibers (MXene concentration: 100 µg mL^−1^) were immersed in the HUVEC suspension (2 × 10^6^ cells mL^−1^) and treated with the NIR laser irradiation (0.40 W cm^−2^, five 2 min ON/OFF cycles). The NIR‐induced temperature was kept around 40 ℃. On days 1 and 3, the cells were stained using a live/dead kit for observation. Cell viability was quantified using the CCK8 kit.

### Scratch Wound‐Healing Assay

HUVECs were seeded in a 24‐well plate (1 × 10^5^ cells/well). After 12 h, a sterile p200 pipette tip was used to scratch the single‐layer cells. The unattached cells were rinsed with PBS twice. Transwell inserts containing MX‐HF scaffolds (MXene concentration: 100 µg mL^−1^) or HF scaffolds (size: 10 mm × 10 mm × 2 mm) were gently transformed into the plate. The cells were photographed at appropriate time points. The wound closure rate was defined as: wound closure rate (%) = (1 – *A*/*A*_0_) × 100%. *A* represents the wound area 15 h after scratching and *A*_0_ represents the wound area immediately after scratching, which were acquired using ImageJ software.

### Matrigel Tube Formation Assay

Matrigel matrix was employed to coat a 24‐well plate (Matrigel:ECM = 1:1; 250 µL per well) before the cell seeding of HUVECs (5 × 10^4^ cells/well). The transwell inserts loaded with MX‐HF scaffolds (MXene concentration: 100 µg mL^−1^) or HF scaffolds (size: 10 mm × 10 mm × 2 mm) were gently placed into the plate. The tube formation of HUVECs was observed after 6 h.

### In Vivo Skin Flap Regeneration

The C57BL/6 mice (male, 22–24 g) randomly grouped (*n*  =  8) as follows: 1) control, 2) HF, 3) MX‐HF, 4) MX‐HF+NIR, 5) VEGF@MX‐HF, and 6) VEGF@MX‐HF+NIR groups. All mice were treated strictly according to the Laboratory Animal Care and Use Guidelines. The experimental protocol was approved by the Animal Care and Use Committee of Wenzhou Medical University (Zhejiang, China). The dorsal hair of mice was shaved one day before wounding. A standard dorsal pedicled flap (size: 3.3 cm × 1.1 cm, length‐to‐width ratio of 3:1) was created and both sacral arteries were sectioned. An HF, MX‐HF or VEGF@MX‐HF scaffold (scaffolds with the size of 3.0 cm × 1.0 cm × 0.5 mm, cropped from a large‐sized scaffolds of 6.0 cm × 6.0 cm × 0.5 mm) was administered hypodermically (Figure 5a–c). For NIR groups, the skin flaps were treated under the exposure of 808 nm laser irradiation at 0.50 W cm^−2^ for five 3 min ON/OFF cycles every three days after implantation. The NIR‐induced temperature was kept around 45 ℃. Random flaps without scaffold implantation and NIR treatment were used as control. On day 9, the blood flow of the skin flap was observed by a Laser Doppler Perfusion Imager (MoorLDI2) and was quantified using the moorLDI and moorBDA systems. The necrotic areas of the flaps were photographed with a digital camera and quantified using ImageJ software. The skin flap necrotic ratio in each group was calculated as the percentages of the necrotic area to the total flap area. For histological analysis, the skin flap specimens around the necrotic junctions were harvested and stained with H&E and Masson's trichrome. Immunofluorescence staining for CD31 (GeneTex) was carried out to analyze angiogenesis efficacy of the scaffolds. Furthermore, TUNEL staining (Beyotime) and immunohistochemical staining for CD163 (HUABIO) were performed to observe the apoptosis and inflammatory response in the skin flaps.

### Statistical Analysis

Data are displayed as means ± standard deviations (*n* ≥ 4). All graphs were created from OriginPro 2020 software. Two‐tailed unpaired Student's *t*‐tests were performed to calculate the statistical significance between two groups, and a *p*‐value < 0.05 was considered significant (denoted as *p < 0.05, **p < 0.01, and ***p < 0.001).

## Conflict of Interest

The authors declare no conflict of interest.

## Author Contributions

Y.J.Z. conceived the idea and designed the experiment; X.C.W. conducted experiments and data analysis; Y.R.Y, C.Y.Y., L.R.S., and X. S. participated in data analysis and discussion. X.C.W. and Y.J.Z. wrote the manuscript.

## Supporting information

Supporting Information

## Data Availability

The data that support the findings of this study are available from the corresponding author upon reasonable request.
