# Supplementary material for: Dynamically Responsive Scaffolds from Microfluidic 3D Printing for Skin Flap Regeneration
Source: Adv Sci (Weinh). 2022 Jun 2;9(22):2201155. doi: 10.1002/advs.202201155 (PMC9353450; doi:10.1002/advs.202201155)
Supplement: Supplementary file 1 — Supporting Information [file ADVS-9-2201155-s001.pdf]

ADVANCED SCIENCE Open Access

Supporting Information

for *Adv. Sci.*, DOI 10.1002/advs.202201155

Dynamically Responsive Scaffolds from Microfluidic 3D Printing for Skin Flap Regeneration

Xiaocheng Wang, Yunru Yu, Chaoyu Yang, Luoran Shang, Yuanjin Zhao* and Xian Shen**

Supporting Information

Dynamically Responsive Scaffolds from Microfluidic 3D Printing for Skin Flap Regeneration

Xiaocheng Wang, Yunru Yu, Chaoyu Yang, Luoran Shang, Yuanjin Zhao* and Xian Shen**

Dr. X. C. Wang, Prof. Y. J. Zhao, and Prof. X. Shen
Department of Gastrointestinal Surgery, The First Affiliated Hospital, Wenzhou Medical University, Wenzhou 325035, China
Email: yjzhao@seu.edu.cn (Y.J.Z.); 13968888872@163.com (X.S.)

Dr. X. C. Wang, Dr. Y. R. Yu, Prof. Y. J. Zhao
Department of Burns and Plastic Surgery, Nanjing Drum Tower Hospital, The Affiliated Hospital of Nanjing University Medical School, Nanjing 210002, China

Dr. X. C. Wang, Dr. Y. R. Yu, Dr. C. Y. Yang, Prof. L. R. Shang, Prof. Y. J. Zhao
Oujiang Laboratory (Zhejiang Lab for Regenerative Medicine, Vision and Brain Health), Wenzhou Institute, University of Chinese Academy of Sciences, Wenzhou, Zhejiang 325001, China

Prof. L. R. Shang
Shanghai Xuhui Central Hospital, Zhongshan-Xuhui Hospital, and the Shanghai Key Laboratory of Medical Epigenetics, the International Co-laboratory of Medical Epigenetics and Metabolism (Ministry of Science and Technology), Institutes of Biomedical Sciences, Fudan University, Shanghai 200032, China.
E-mail: luoranshang@fudan.edu.cn

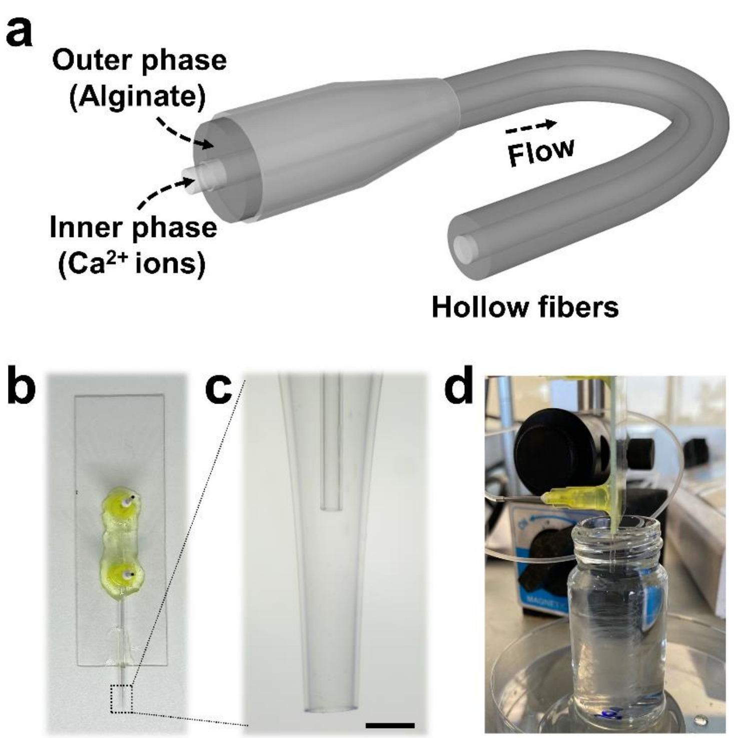

Figure S1. (a) Schematic illustration of the microfluidic spinning of the hollow microfibers. (b, c) Photographs of the custom-made coaxial capillary microfluidic chip with a spindle capillary (orifice diameter: 100 μm) and a tapered capillary (orifice diameter: 450 μm). Scale bar, 500 μm. (d) Photographs of hollow microfibers collected in a vessel during microfluidic spinning.

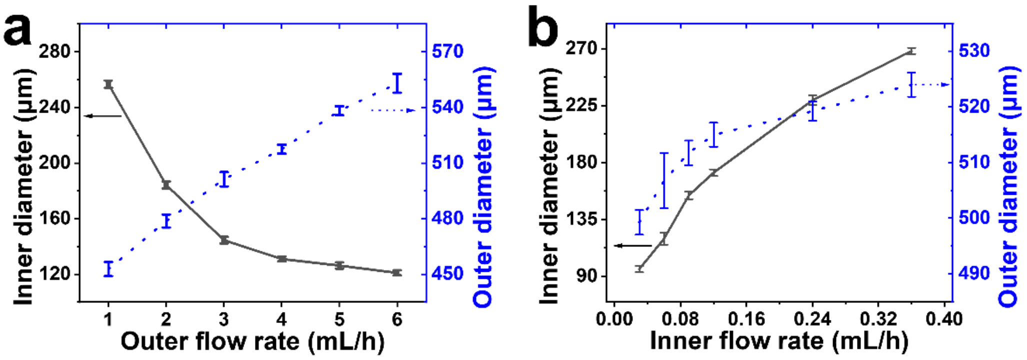

Figure S2. (a-b) Relationships between the inner and outer diameter of the hollow microfibers and the flow rates of the (a) outer and (b) inner phases. The arrows indicate the Y-axis for the relative lines (n = 6 per group).

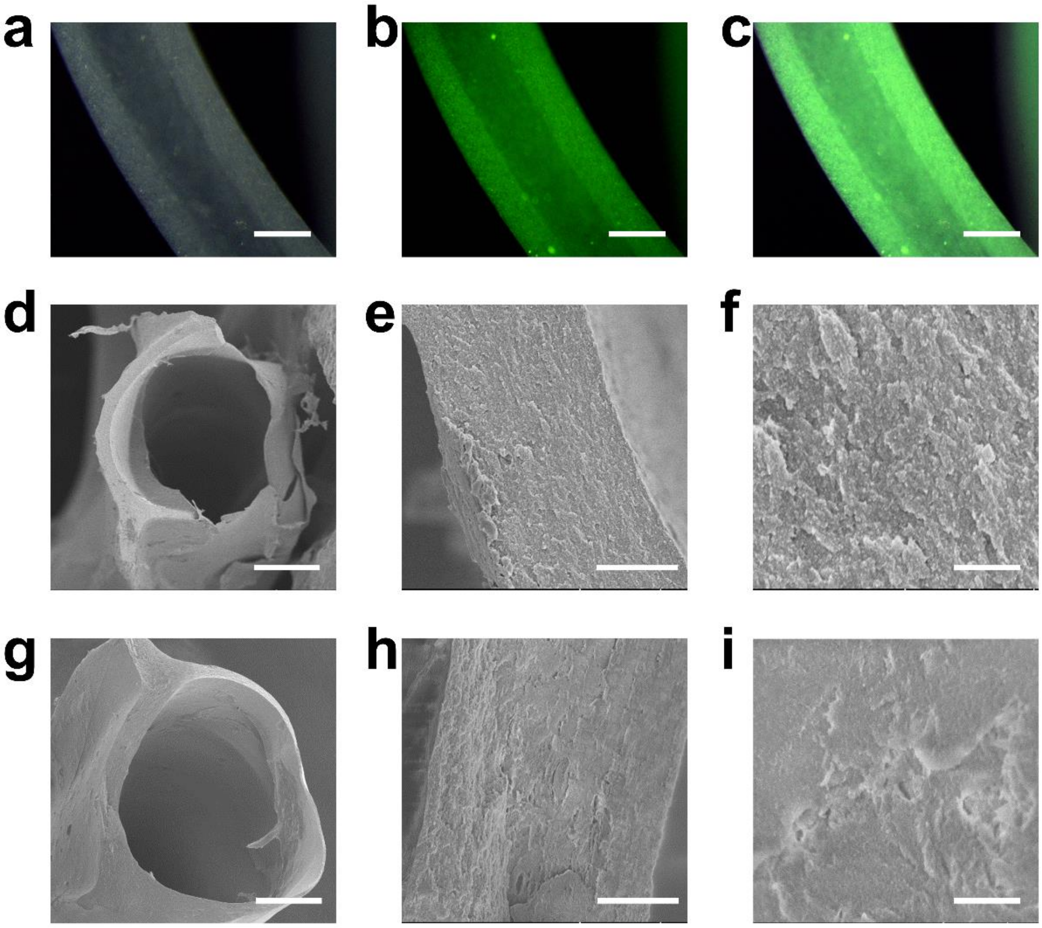

Figure S3. (a-c) Optical and fluorescent micrographs of the hollow microfibers containing green fluorescent nanoparticles of 501/515 nm. Sectional views of SEM morphologies of (d-f) the hollow microfibers and (g-i) MXene-containing hollow microfibers at different magnifications. The scale bars indicate 200 μm in (a-c), 50 μm in (d, g), 5 μm in (e, h), and 1 μm in (f, i).

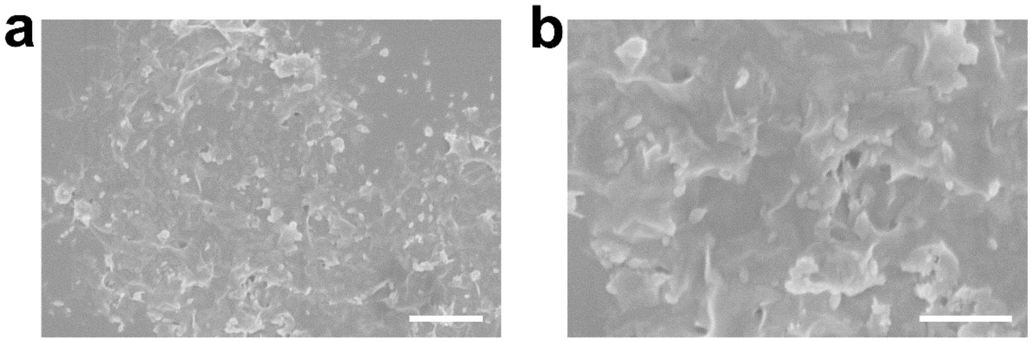

Figure S4. SEM morphologies of MXene nanosheets at different magnifications. The scale bars indicate 1 μm in (a), and 500 nm in (b).

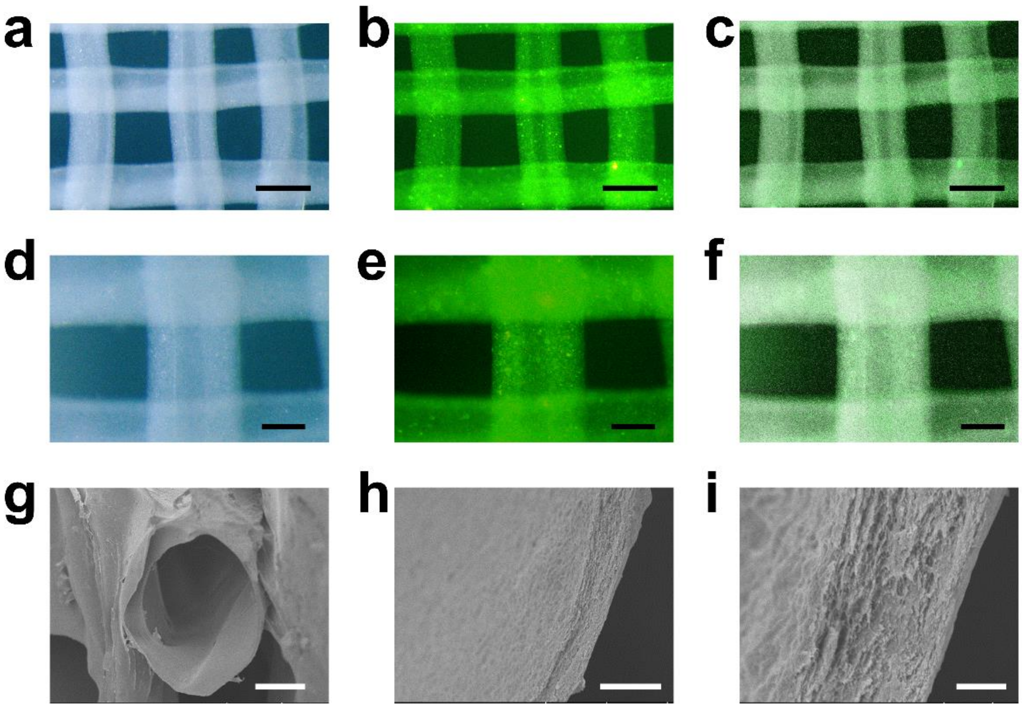

Figure S5. (a-f) Optical and fluorescent micrographs of the hollow fibrous (HF) scaffolds containing green fluorescent nanoparticles at different magnifications. (g-i) Sectional views of SEM morphologies of the HF scaffolds at different magnifications. The scale bars indicate 500 μm in (a-c), 200 μm in (d-f), 50 μm in (g), 5 μm in (h), and 1 μm in (i).

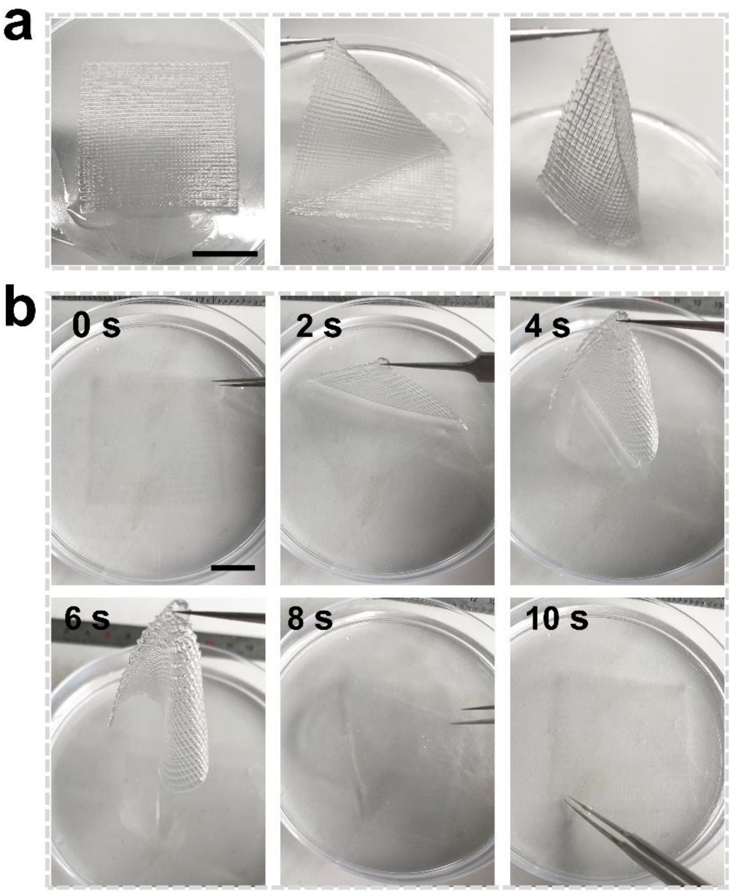

Figure S6. Photographs of the MXene-containing hollow fibrous (MX-HF) scaffolds with 6.0 cm (width) × 6.0 cm (length) × 0.5 mm (height) in the (a) dry and (b) wet Petri dishes. The scaffolds had sufficient mechanical properties to be picked up (a) and easily unfolded using a tweezer within 10s in liquids (b). Scale bar, 2 cm.

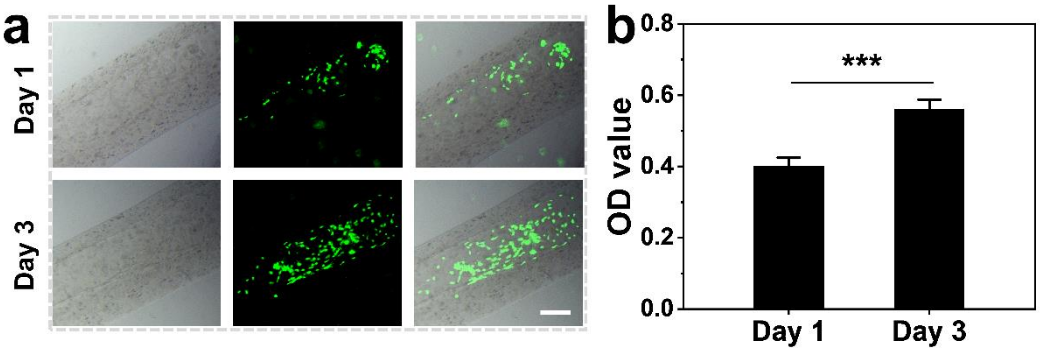

Figure S7. Fluorescent images of human umbilical vein endothelial cells (HUVECs) enriched in the MX-HF scaffold channels and cultured for 3 days. The scale bar indicates 200 μm. n = 6 per group, two-tailed unpaired Student's t-tests were performed to calculate the statistical significance between two groups, and *$p < 0.05$, **$p<0.01$, and ***$p<0.001$.

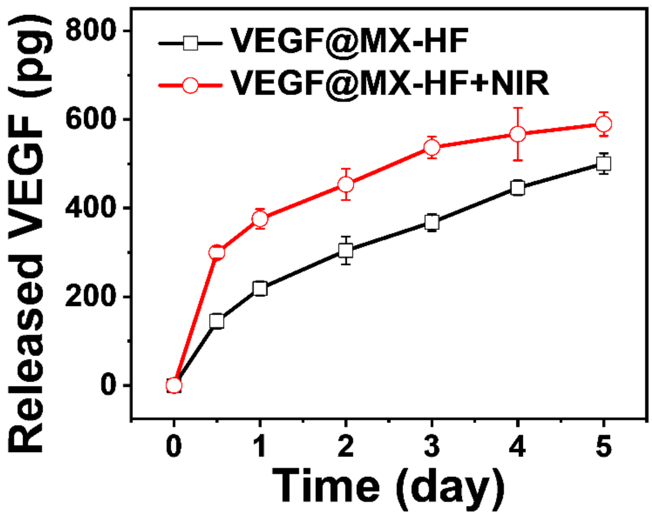

Figure S8. The VEGF release profiles of the VEGF@MX-HF scaffolds with or without NIR irradiation (808 nm, 0.40 W/cm^2, 3 min; n = 6 per group).

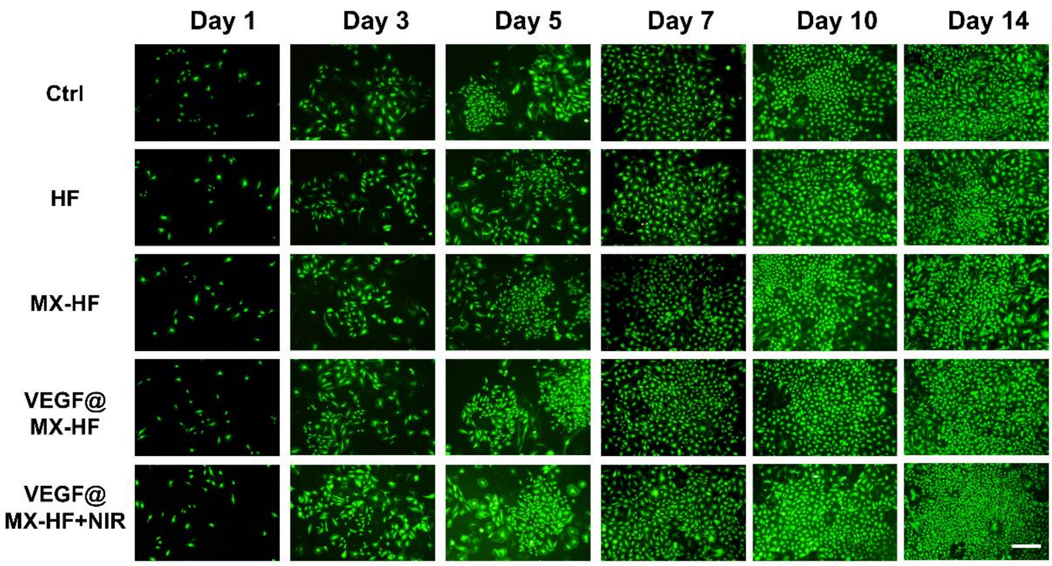

Figure S9. Live/dead staining images of HUVECs cultured with HF, MX-HF or VEGF@MX-HF scaffolds for 14 days with or without NIR irradiation (0.40 W/cm^2, 3 min, Once a day). Alive or dead cells were in green or red, respectively. The scale bar indicates 500 μm.

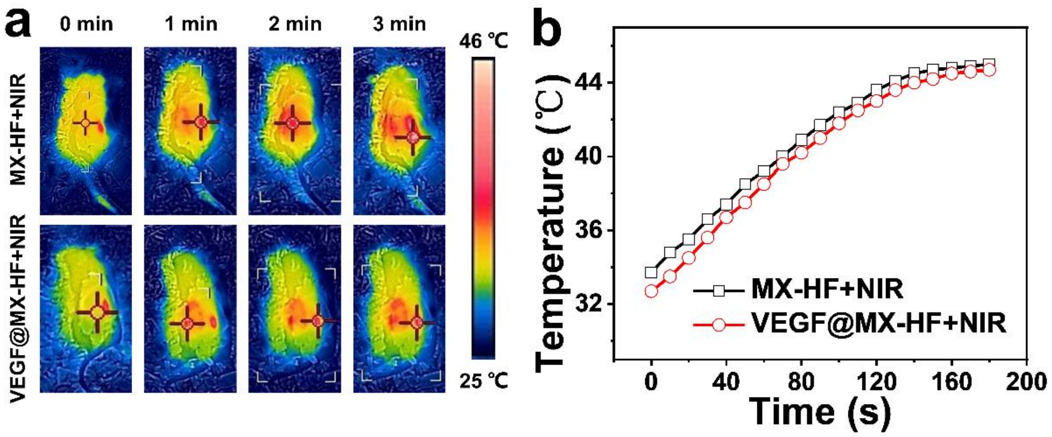

Figure S10. (a) Real-time thermal images and (b) corresponding photothermal heating curves of skin flaps post-implanted with of MX-HF and VEGF@MX-HF scaffolds under the irradiation of an 808-nm laser at 0.50 W/cm^2 for 3 min.

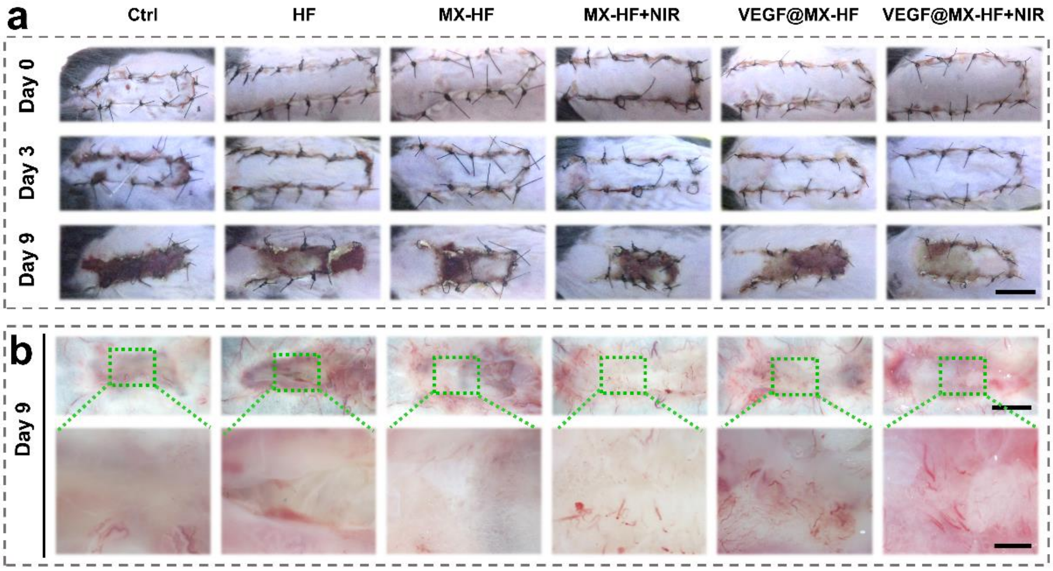

Figure S11. (a) Digital photographs of the flaps on days 0, 3, and 9 post surgery. (b) Optical images showing the angiogenesis states of the detached flaps on day 9 from different groups. Scale bars indicate 1 cm in (a), 5 mm in (b, top), and 1 mm in (b, bottom).

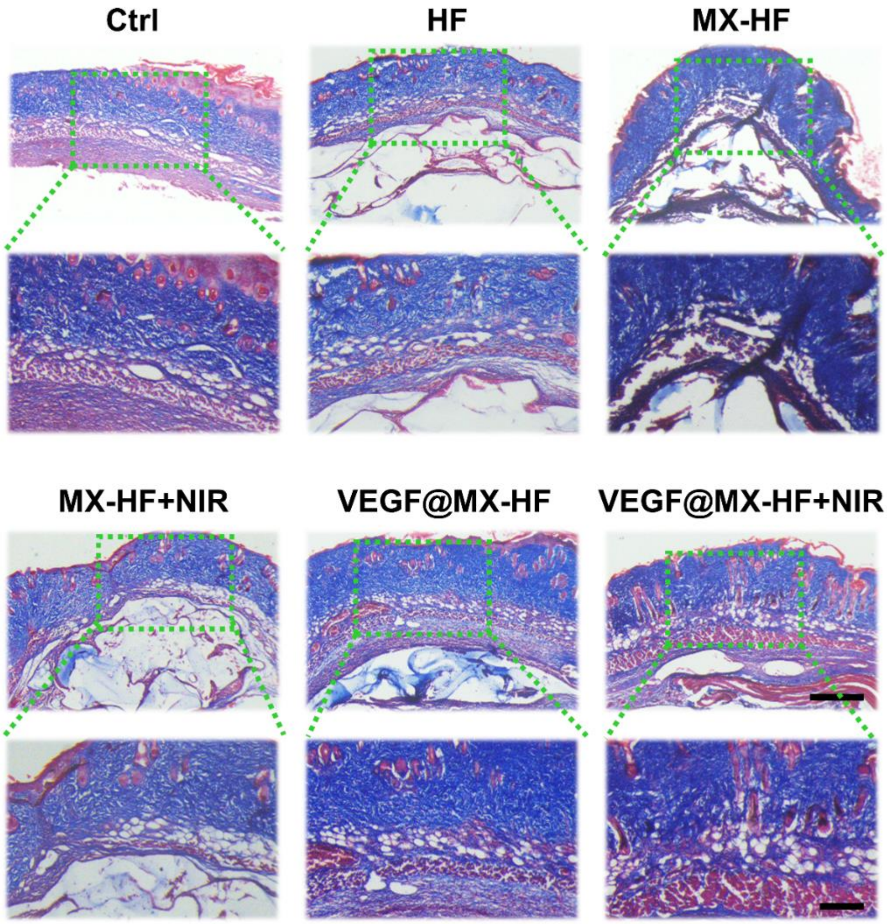

Figure S12. Masson staining of the necrosis and survival junction area of the skin flaps in different groups. Scale bars indicate 500 μm (top) and 200 μm (bottom).
